# The significance of central blood pressure for cardiovascular target organ damage in children and adolescents after kidney transplantation

**DOI:** 10.1007/s00467-022-05857-y

**Published:** 2023-01-11

**Authors:** Anne-Sophie Greiner, Jeannine von der Born, Lena Kohlmeier, Carl Grabitz, Elena Bauer, Nima Memaran, Rizky Indrameikha Sugianto, Nele Kanzelmeyer, Kerstin Fröde, Bernhard Schmidt, Anette Melk

**Affiliations:** 1 Department of Pediatric Kidney, Liver and Metabolic Diseases, Hannover Medical School, Carl-Neuberg-Str. 1, 30625 Hannover, Germany; 2 Department of Nephrology and Hypertension, Hannover Medical School, Hannover, Germany

**Keywords:** Children, Kidney transplantation, Central blood pressure, LVMI, Pulse wave velocity

## Abstract

**Background:**

Cardiovascular (CV) complications are important causes of morbidity and mortality in children after kidney transplantation (KTx). In adults, central blood pressure (cBP) is an accepted predictor of CV sequelae. We aimed to assess the prognostic value of cBP over peripheral blood pressure (pBP) for existing CV damage.

**Methods:**

We measured cBP and pBP in 48 pediatric KTx recipients (mean age: 13.5 ± 4.2 years). Assessment of left ventricular mass index (LVMI) and aortic pulse wave velocity (PWV) allowed detection of CV target organ damage. LVMI and PWV were used as endpoints in multivariable linear regression models, in which cBP and pBP were compared for their predictive value.

**Results:**

Using cBP z-scores, we identified a larger number of patients with uncontrolled or untreated hypertension compared to pBP (36% vs. 7%). Central systolic blood pressure (cSBP) was a significant independent predictor of LVMI, while peripheral systolic blood pressure (pSBP) was not. Comparing central (cDBP) and peripheral (pDBP) diastolic blood pressure for their predictive value on PWV revealed a greater estimate for cDBP (0.035 vs. 0.026 for pDBP) along with a slightly better model fit for cDBP.

**Conclusions:**

Our data in a small group of patients provide first evidence that cBP measurements in pediatric KTx recipients might be helpful in identifying patients at risk for the development of CV sequelae. Investigating a larger patient number, ideally repeatedly, is needed to create further evidence supporting our findings. In light of available devices measuring cBP noninvasively, the implementation of such clinical studies post-KTx care should be feasible.

**Graphical abstract:**

**Figure:**
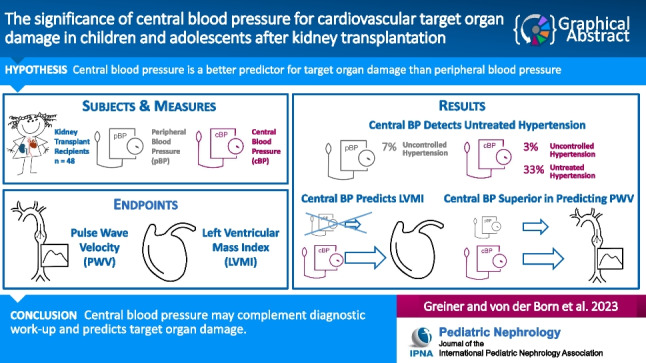
A higher resolution version of the Graphical abstract is available as Supplementary information

**Supplementary Information:**

The online version contains supplementary material available at 10.1007/s00467-022-05857-y.

## Introduction

Children after kidney transplantation (KTx) suffer from a high cardiovascular (CV) burden and CV events are still among the most important causes of morbidity and mortality in these patients [1, 2]. Early vascular damages resembling athero- and arteriosclerotic processes have been detected by measuring carotid intima-media thickness and aortic pulse wave velocity (PWV) in this high-risk population [3–7]. Similarly, left ventricular hypertrophy (LVH) indicates early cardiac alterations and is frequently detected [3, 8]. These easy-to-measure parameters are well-accepted surrogate markers and have been shown to be highly predictive for future CV events in adult patients [9].

The prevalence of arterial hypertension is high in pediatric KTx recipients [3, 10]. It is not only a major risk factor for CV alterations [4, 11], but also negatively affects graft function [12, 13]. While arterial hypertension is a modifiable risk factor, blood pressure (BP) control in children after KTx remains difficult and needs to be optimized as reported in several recent studies [3, 10, 14, 15]. In addition, relying on peripheral blood pressure (pBP) may not be ideal. Studies in adults showed that central blood pressure (cBP) correlates better with the severity of CV alterations and is a better predictor of CV mortality [16, 17]. In hypertensive children and in children with type 1 diabetes, cBP showed good correlations with PWV [18, 19] and the prevalence of LVH [20]. The implementation of cBP measurements could therefore be helpful to identify high-risk patients. New devices such as the Mobil-O-Graph, which has been validated in children against invasive cBP measurements [21], allow non-invasive and easy-to-perform cBP measurements.

As no data for children after KTx currently exist, we aimed to examine the predictive value of cBP over pBP for existing CV damage detected by measurement of PWV and left ventricular mass index (LVMI) in pediatric KTx recipients. We used the Mobil-O-Graph as it not only has been validated against invasive recordings in children and adults [21, 22], but also because reference data with z-scores is available for children [23].

## Methods

### Study population

For this prospective study, we examined a total of 48 children, adolescents, and young adults, who received KTx before the age of 18 years. We included only patients with a functioning graft. Patient data (anthropometrics, underlying disease, transplantation history, dialysis prior to transplantation) and current medication information were obtained from the medical charts. Blood samples were obtained and analyzed in a central laboratory (Synlab, Heidelberg, Germany). The analysis included creatinine, cystatin C, and urea. Estimated glomerular filtration rate (eGFR) was calculated as proposed by Schwartz et al. (0.41 × height (cm)/plasma-creatinine (mg/dl)) [24]. Z-scores for height, weight [25], and waist circumference [26] were calculated. Overweight was defined as BMIz ≥ 1.036 (≥ 85^th^ percentile) and < 1.645 (< 95^th^ percentile) of sex-specific z-score range; obesity was defined as BMIz ≥ 1.645 (95^th^ percentile) age and sex-specific z-score [27]. The study protocol was approved by the institutional review board (No. 504–2009) and all parents and children gave informed consent.

### Blood pressure

BP measurements were performed as follows: pBP was measured using a validated oscillometric device (Dinamap v100, GE Medical System) after 5 min of rest in a seated position on the right and left arm, one measurement each. pBP z-scores (pBPz) were calculated according to the reference values from the National High Blood Pressure Education Program Working Group in Children and Adolescents [28]. cBP was measured using the Mobil-O-Graph (IEM: GmbH, Stollberg), a validated oscillometric device [21, 22]. Following the instructions of the manufacturer and previous reports [21] cBP was measured after a resting period of at least 5 min in a spinal position. The mean of three consecutive measurements taken on the right arm was used. The device’s software offers two different calibration (C) methods: C1 is based on the measurement of peripheral systolic (pSBP) and diastolic BP (pDBP) with the advantage of existing reference data for central systolic blood pressure (cSBP) values in children aged ≥ 8 years or ≥ 112 cm in girls or ≥ 123 cm in boys, respectively. Values are expressed as z-scores (cSBPz) adjusted for sex and either height or age [23]. C2 is based on the mean arterial BP and the peripheral diastolic BP (DPB) and is considered to provide better accuracy for cBP [29, 30], but offers no reference data. We report cBP based on both calibration methods for this study. We defined pBP values as elevated if the z-score for systolic BP (pSBPz) or diastolic BP (pDBPz) was ≥ 1.645 (95^th^ percentile) [31]. Similarly, cSBP-C1 values with a z-score ≥ 1.645 (95^th^ percentile) were considered elevated. Further classification of elevated BP also considered the use of antihypertensive medication. We defined patients on antihypertensive medication displaying elevated BP values as having “uncontrolled hypertension,” while those with normotensive BP were classified as “controlled hypertension.” Patients who did not receive antihypertensive drugs but displayed elevated BP values were classified as having “untreated hypertension.”

### Pulse wave velocity, PWV

PWV was measured using the oscillometric Vicorder device (Skidmore Medical Limited, Bristol, UK; software version 8.3.7244.18754) following a standardized protocol in accordance with the current guidelines [32] as reported previously [33]. The measured velocities were expressed as absolute values and z-scores standardized to height (PWVz) [33].

### Echocardiography

Transthoracic echocardiography was performed using a Philips CX 50 ultrasound device (Philips Medical System, Amsterdam, Netherlands), equipped with a 1–5-MHz transducer, according to the recommendations of the American Society of Echocardiography [34]. All studies were evaluated by a single experienced investigator. Wall thickness and dimensions of the left ventricle were measured in the parasternal short axis view at the level of the papillary muscles using M-mode. LVMI was calculated as proposed by Chinali et al. [35]. Children with a LVMI > 45/m^2.16^ were considered to have LVH.

### Methods against bias

pBP and cBP were measured sequentially by a study nurse or a trained medical student. The devices were set to measure BP with mentioned repeats and results were stored in the devices’ data cache until all examinations were performed. Measurements were then transferred to the study’s case report form. Echocardiography was performed and evaluated by a single pediatric cardiologist. PWV was measured by two experienced physicians. None of the physicians was aware of the blood pressure examinations.

### Statistical analysis

Data are given as mean ± standard deviation (SD) or frequency and percentage. Paired *t*-test was used for paired comparison of cBPz and pBPz and paired ANOVA for cBP-C1, cBP-C2, and pBP. Multiple comparisons were corrected with the Tukey adjustment. Multivariable linear regression analyses for LVMI and PWV were performed to investigate the effect of pBP, cBP-C1, and cBP-C2, adjusted for sex, age [10, 33, 36], and eGFR [13, 37, 38]. The goodness of fit, *R*^2^, was considered for the comparison of predictive values between cBP-C1, cBP-C2, and pBP on LVMI or PWV. A two-tailed *p*-value of < 0.05 was considered statistically significant. Statistical analysis was performed using the SAS Enterprise Guide 7.1 (Statistical Analysis Software, Cary, NC, USA).

## Results

### Patient characteristics

We included 48 pediatric KTx recipients in the study. Mean age was 13.5 ± 4.2 years (range 5–24 years), 22 (46%) were girls, mean height was 151.3 ± 21.2 cm (z-score − 0.4 ± 1.3), and mean BMI was 20.7 ± 4.3 kg/m^2^ (z-score 0.3 ± 1.0). Congenital anomalies of the kidney and urinary tract (CAKUT) as the underlying disease was found in 32 patients (67%). Mean time since last transplantation was 5.5 ± 4.6 years, 20 patients (42%) were transplanted preemptively, and 4 (8%) received a re-transplantation. Mean eGFR was 76.8 ± 37.5 ml/min/1.73 m^2^. The majority of patients (*n* = 44, 92%) received a calcineurin inhibitor–based immunosuppression (cyclosporine A, *n* = 31; tacrolimus, *n* = 13) in combination with either mammalian target of rapamycin (mTOR) inhibitors (*n* = 39, 81%) or mycophenolate mofetil (MMF; *n* = 8, 17%). Twenty-three (48%) patients were on steroids.

### BP measurements

Table 1 provides pBP or cBP assessments as absolute and standardized values.

**Table 1:** Absolute and standardized values for peripheral (pBP) and central (cBP) blood pressure. For cBP, values according to the two available calibration methods (C1 and C2) are provided

	*N*	Mean ± SD
Peripheral blood pressure		
pSBP		
Absolute (mmHg)	48	113.2 ± 10.5
Standardized (z-score, sex, age & height)	48	0.5 ± 0.8
pDBP		
Absolute (mmHg)	48	67.0 ± 10.3
Standardized (z-score, sex, age & height)	48	0.4 ± 1.0
Central blood pressure-C1		
cSBP-C1		
Absolute (mmHg)	48	107.4 ± 8.8
Standardized (z-score, sex & height)	42	1.3 ± 1.3
Standardized (z-score, sex & age)	40	0.8 ± 1.1
cDBP-C1		
Absolute (mmHg)	48	73.3 ± 9.0
Central blood pressure-C2		
cSBP-C2		
Absolute (mmHg)	47	116.5 ± 13.4
cDBP-C2		
Absolute (mmHg)	47	72.7 ± 9.1

*BP*, blood pressure; *cDBP-C1*, central diastolic blood pressure-calibration 1; *cDBP-C2*, central diastolic blood pressure-calibration 2; *cSBP-C1*, central systolic blood pressure-calibration 1; *cSBP-C2*, central systolic blood pressure-calibration 2; *pSBP*, peripheral systolic blood pressure; *pDBP*, peripheral diastolic blood pressure

#### pBP

Mean pSBP and pDBP were 113.2 ± 10.5 mmHg and 67.0 ± 10.3 mmHg, respectively. Five children (10%) had elevated pSBP, and three children (6%) had elevated pDBP values.

#### cBP

**Fig. 1:**
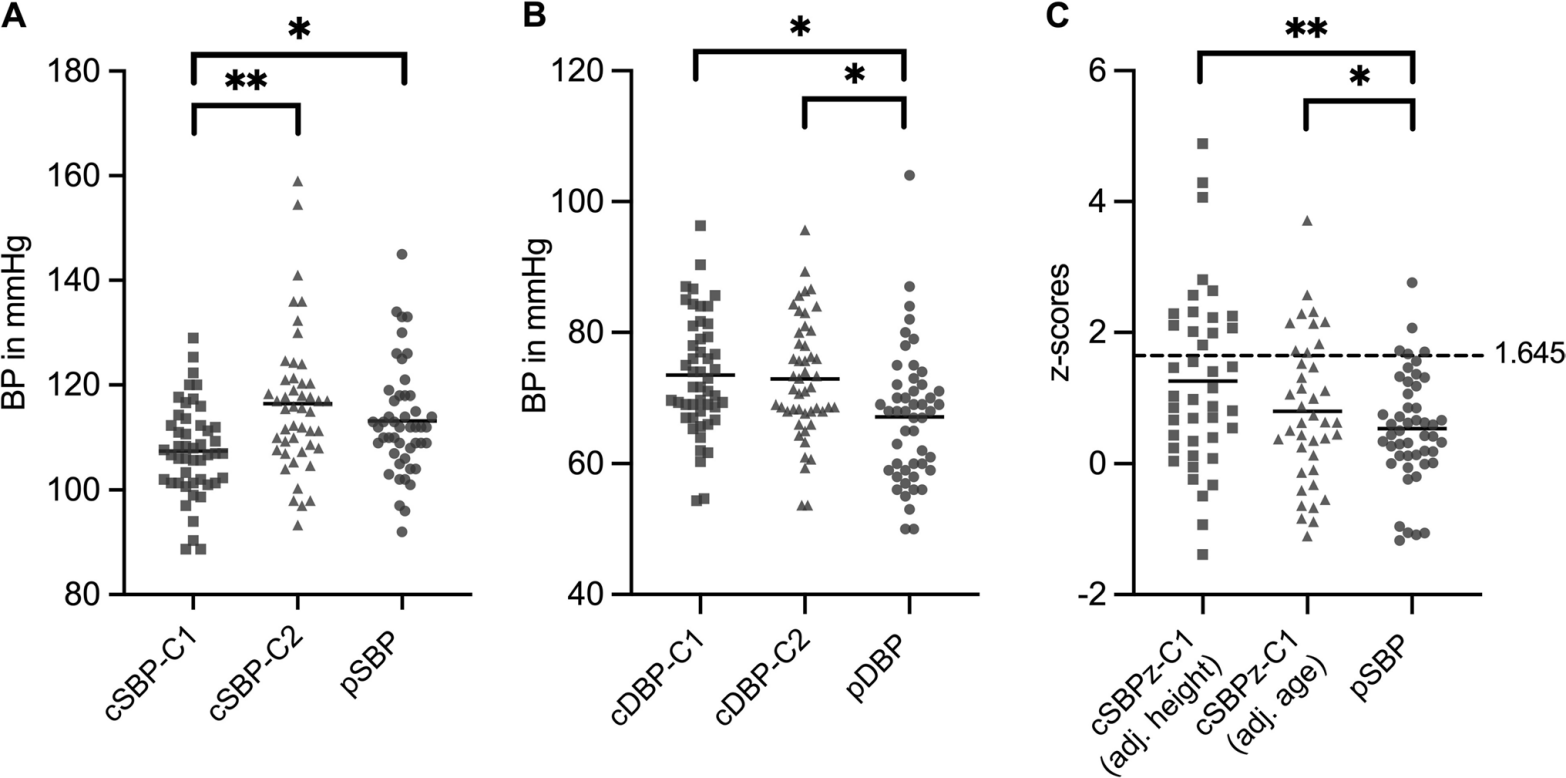
Comparison of central (cBP) and peripheral (pBP) blood pressure. (**A**) Absolute systolic blood pressure (SBP) values based on two different calibration methods (C1 and C2) for cBP (cSBP-C1 and cSBP-C2) compared to pSBP. (**B**) Absolute diastolic blood pressure (DBP) values comparing cDBP-C1, cDBP-C2, and pDBP. (**C**) Z-scores for SBP comparing cSBPz-C1 adjusted for either height or age with pSBPz (adjusted for sex, age, height). Data are presented as absolute values or z-scores, respectively. Bars indicate the mean. The dotted line marks a z-score of 1.645. “*” indicates a significance of *p* < 0.05; “**” indicates a significance of < 0.001

The Mobil-O-Graph provides C1 and C2 values based on two calibration methods. Mean cSBP-C1 and cDBP-C1 were 107.4 ± 8.8 mmHg and 73.3 ± 9.0 mmHg, while mean cSBP-C2 and cDBP-C2 were 116.5 ± 13.4 mmHg and 72.7 ± 9.1 mmHg. As illustrated in Fig. 1A, cSBP-C2 was significantly higher than cSBP-C1 (*p* < 0.001); for diastolic absolute values, there was no significant difference as presented in Fig. 1B.

#### Comparing cBP and pBP

SBP absolute values for cSBP-C1 were significantly lower than pSBP (*p* = 0.03), with no difference between cSBP-C2 and pSBP (Fig. 1A). DBP values for either cDBP-C1 or cDBP-C2 were both significantly higher compared to pDPB (*p* = 0.004 and *p* = 0.01; Fig. 1B). The two different cSBP-C1 z-scores (cSBPz-C1) adjusted for sex and either height or age were significantly higher than the pSBPz (*p* < 0.001 and *p* = 0.03) (Fig. 1C).

**Fig. 2:**
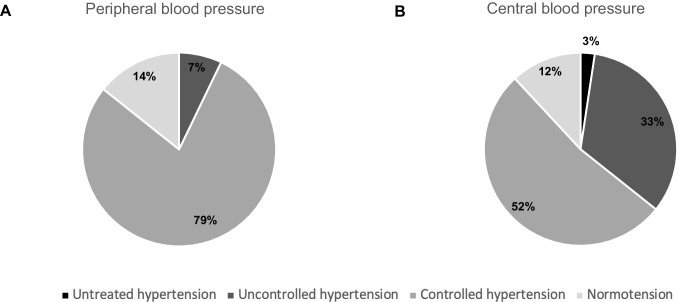
Prevalence of hypertension based on (**A**) peripheral or (**B**) central blood pressure values. Classification is based on elevated SBP values according to z-scores and the use of antihypertensive medication

Based on SBP z-scores from pBP and cBP measurements and the use of antihypertensive medication, we aimed to group patients as being normotensive or having untreated, uncontrolled, or controlled hypertension. Based on pSBPz, we identified three (7%) patients having uncontrolled hypertension, 33 (79%) with controlled hypertension, and six (14%) had normotensive pBP values (Fig. 2A). Using cSBPz-C1, we identified 14 (33%) patients with uncontrolled hypertension, 22 (52%) with controlled hypertension, and five (12%) displayed normotensive cSBP-C1 values (Fig. 2B). In addition, one child (3%) was identified with untreated hypertension.

### CV damage

As higher BP is known to cause CV damage, we evaluated the effect of pBP and cBP on LVMI and PWV, which we had measured in the majority of patients from our cohort.

#### LVMI

LVMI values were available for 47 patients. Mean LVMI was 38.3 ± 11.0 g/m^2.16^. Left ventricular hypertrophy was found in ten (21%) patients. We composed a multivariable linear regression model predicting LVMI and adjusted for sex, age, eGFR, and pSBP (Table 2). While we found a tendency for lower eGFR to affect LVMI, pSBP had no effect on LVMI. To evaluate whether either cSBP-C1 or cSBP-C2 would have predictive value for LVMI, we replaced pSBP in two respective models. While the introduction of cSBP-C1 revealed no significant effect on LVMI, cSBP-C2 showed a significant association with LVMI (estimate for cSBP-C2: 0.326, *p* = 0.04). In addition, the model including cSBP-C2 showed a superior fit (*R*^2^ = 0.182) when compared to the model including either pSBP (*R*^2^ = 0.156) or cSBP-C1 (*R*^2^ = 0.112).

**Table 2:** Comparison of cBP and pBP using a multivariable linear regression model

	Parameters	*β*	CI 95%	*p*-value	Parameters	*β*	CI 95%	*p*-value	Parameters	*β*	CI 95%	*p*-value
LVMI	Intercept	8.115			Intercept	21.64			Intercept	6.288		
	Sex (reference male)	0.554	− 6.30; 7.41	0.87	Sex (reference male)	− 0.232	− 7.51; 7.05	0.95	Sex (reference male)	2.385	− 5.3; 10.07	0.53
	Age	0.122	− 0.75; 0.99	0.78	Age	0.284	− 0.66; 1.22	0.55	Age	− 0.066	− 0.98; 0.84	0.88
	eGFR	− 0.08	− 0.17; 0.01	0.07	eGFR	− 0.090	− 0.18; 0.001	0.05	eGFR	− 0.083	− 0.17; 0.004	0.06
	pSBP	0.306	− 0.06; 0.67	0.10	cSBP-C1	0.184	− 0.27; 0.64	0.42	cSBP-C2	0.326	0.01; 0.64	0.04
	Model *R*^2^ 0.156				Model *R*^2^ 0.112				Model *R*^2^ 0.182			
PWV	Intercept	3.133			Intercept	2.664			Intercept	2.579		
	Sex (reference male)	− 0.244	− 0.62; 0.13	0.20	Sex (reference male)	− 0.347	− 0.7; 0.01	0.05	Sex (reference male)	− 0.345	− 0.71; 0.01	0.06
	Age	0.071	0.03; 0.11	0.002	Age	0.053	0.01; 0.10	0.02	Age	0.049	0.01; 0.10	0.04
	eGFR	− 0.002	− 0.01; 0.003	0.40	eGFR	− 0.002	− 0.01; 0.003	0.50	eGFR	− 0.001	− 0.01; 0.003	0.55
	pDBP	0.026	0.01; 0.04	0.005	cDBP-C1	0.033	0.01; 0.06	0.003	cDBP-C2	0.035	0.01; 0.06	0.003
	Model *R*^2^ 0.453				Model *R*^2^ 0.462				Model *R*^2^ 0.469			

*cDBP-C1*, central diastolic blood pressure-calibration 1; *cDBP-C2*, central diastolic blood pressure-calibration 2; *cSBP-C1*, central systolic blood pressure-calibration 1; *cSBP-C2*, central systolic blood pressure-calibration 2; *eGFR*, estimated glomerular filtration rate; *pDBP*, peripheral diastolic blood pressure; *pSBP*, peripheral systolic blood pressure

#### PWV

PWV values were available for 43 patients. Mean absolute values for PWV were 5.6 ± 0.7 m/s, and the mean PWVz was 1.1 ± 1.1. Sixteen (37%) patients had an elevated PWVz.

Similar to what we had done for LVMI, we composed a multivariable linear regression model predicting PWV adjusted for sex, age, eGFR, and either pSBP or pDPB. We showed that higher pDBP was significantly and independently associated with higher PWV, while pSBP was not. Therefore, we used pDBP for the remaining analysis. Age, but neither sex nor eGFR, was an independent predictor of PWV. Introduction of either cDBP-C1 or cDBP-C2 revealed that both values were independently associated with higher PWV. We found higher estimates for cDBP-C1 and cDBP-C2 (0.033 and 0.035) compared to pDBP (0.026) along with a slightly better model fit for the cDBP-C1 and cDBP-C2 (*R*^2^ = 0.462 and *R*^2^ = 0.469 vs. *R*^2^ = 0.453; Table 2).

## Discussion

We evaluated cBP compared to pBP in a cohort of pediatric KTx recipients with a rather high CV burden. We found not only a higher frequency of elevated cSBPz-C1 values when compared to pSBPz, we also found that cBP-C2 was superior over pBP in predicting LVMI and PWV, both important parameters reflecting CV target organ damage. While further investigations with larger patient numbers and longitudinal assessments are surely needed, our data suggest that a larger number of patients than expected has to be considered at risk for CV morbidity and potentially mortality and should be treated preventively. In light of the easy-to-use cBP devices, the implementation of routine cBP measurements into clinical practice to gain more insight into the CV health of pediatric KTx recipients would be feasible.

Our finding that cSBP-C2 was superior in predicting LVMI when compared to pSBP using a multivariable linear regression model extends previous findings, which showed similar associations but were limited to univariate approaches. A possible superiority of cSBP compared to pSBP in predicting LVM has been demonstrated in cohorts of adolescents with untreated hypertension compared to healthy peers [18, 20]. Using the Mobil-O-Graph device, Ntineri et al. demonstrated a higher sensitivity of cSBP-C2 compared to pSBP in predicting LVMI using simultaneous 24-h peripheral and central ambulatory BP monitoring in individuals referred for suspected hypertension and healthy volunteers [20]. Comparable results using the Vicorder device and reference data derived from adults were published by Litwin et al. [18]. While multivariable linear regression models revealed no predictive value of cSBP for LVMI, a ROC analysis showed that cSBP had a greater predictive power for LVH than 24-h ambulatory SBP. Another study in obese children using the Mobil-O-Graph [39] found cBP to be higher in overweight children with and without hypertension. Higher cBP correlated with higher LVMI and PWV, but only in a univariate analysis. A study of 46 adult patients after kidney transplantation showed a significant association between cSBP and LVMI, again only in a univariate approach [40]. The fact that our patient cohort had a considerably high CV burden compared to the other studies discussed here may have allowed us to work out the importance of cSBP in this specific patient group.

We found that in our cohort cDBP-C2 over pDBP could be a better predictor of a higher PWV, which is an important indicator of early vascular damage. Although pDBP and cDBP were both relevant predictors of PWV, we saw a slightly better model fit using cDBP-C2. The few studies addressing this question come up with controversial results. While some studies reported no differences between cBP and pBP in their association with PWV [20, 41], other authors did [17–19]. Tagetti et al. [19] showed in a large cohort of children with type 1 diabetes that cSBP and cSBPz were significant predictors of PWV/PWVz in multivariable regression analysis adjusted for age, sex, and different diabetes-specific parameters. This study also demonstrated a superiority of cBP over pBP. In a large cohort of healthy children, Peluso et al. [17] demonstrated a stronger correlation of cBP compared to pBP with vascular alterations such as PWV, but this analysis was only univariate and the predictive value of cDBP over pDBP for higher PWV was not addressed. The study by Litwin et al. [18] also evaluated PWV and found an association of elevated cSBP with higher values for PWV-SDS.

Not only did we show the importance of cBP measurements in predicting CV damage, we also found an unexpected high prevalence of uncontrolled and untreated hypertension using cBP in our cohort of KTx recipients. While the latter could only be determined by using cSBPz-C1 levels, we must assume the results would have been similar if z-scores for cSBP-C2 values were available. Several recent studies reported a high prevalence of uncontrolled or untreated hypertension based on pBP measurements in pediatric patients after KTx [3, 10, 42], but data considering cBP in this particular high-risk group are not available yet. Previous studies used applanation tonometry (SphygmoCor) as an established method for estimating cBP. A comparison of the oscillometric device (Mobil-O-Graph) used in this study with the Sphygmocor revealed no significant differences for the cBP estimation and comparable reproducibility [43]. In light of arterial hypertension being a major but modifiable risk factor associated with CV complications and poor long-term prognosis in children and adolescents after KTx [2, 12, 13], a greater sensitivity in detecting patients with elevated BP in combination with an easy-to-use device will allow us to provide earlier treatment and should consecutively lower patient risk for a poor outcome.

Limitations of our study are the cross-sectional approach and the small sample size. The high prevalence of early CV alterations still enabled us to provide insight into the predictive capacity of cBP and thereby the clinical relevance of these findings in our particular cohort. Further longitudinal assessment within a larger group of patients is required to confirm our results.

## Conclusion

While in adults the prognostic value of cBP had been repeatedly reported [16, 17], the predictive power of cBP in context of CV disease in children, especially after KTx, had not been studied. With our data, we provide the first evidence that cBP measurements in pediatric KTx recipients could be helpful in identifying patients at risk for the development of important CV sequelae. Studies investigating a larger number of patients at multiple time points are needed to further support our findings on the predictive capacity of cBP in children. In light of the availability of devices capable of measuring cBP noninvasively and with only minimal training, the implementation of such clinical studies post-KTx care should be feasible.

## Supplementary Information

Below is the link to the electronic supplementary material.

Graphical Abstract (PPTX 264 KB)

## Data Availability

Data available upon reasonable request to the corresponding author.
