# Supplementary material for: The significance of central blood pressure for cardiovascular target organ damage in children and adolescents after kidney transplantation
Source: Pediatr Nephrol. 2023 Jan 11;38(8):2791–9. doi: 10.1007/s00467-022-05857-y (PMC10393860; doi:10.1007/s00467-022-05857-y)
Supplement: Supplementary file 1 — Graphical Abstract (PPTX 264 KB) [file 467_2022_5857_MOESM1_ESM.pptx]

## Slide 1
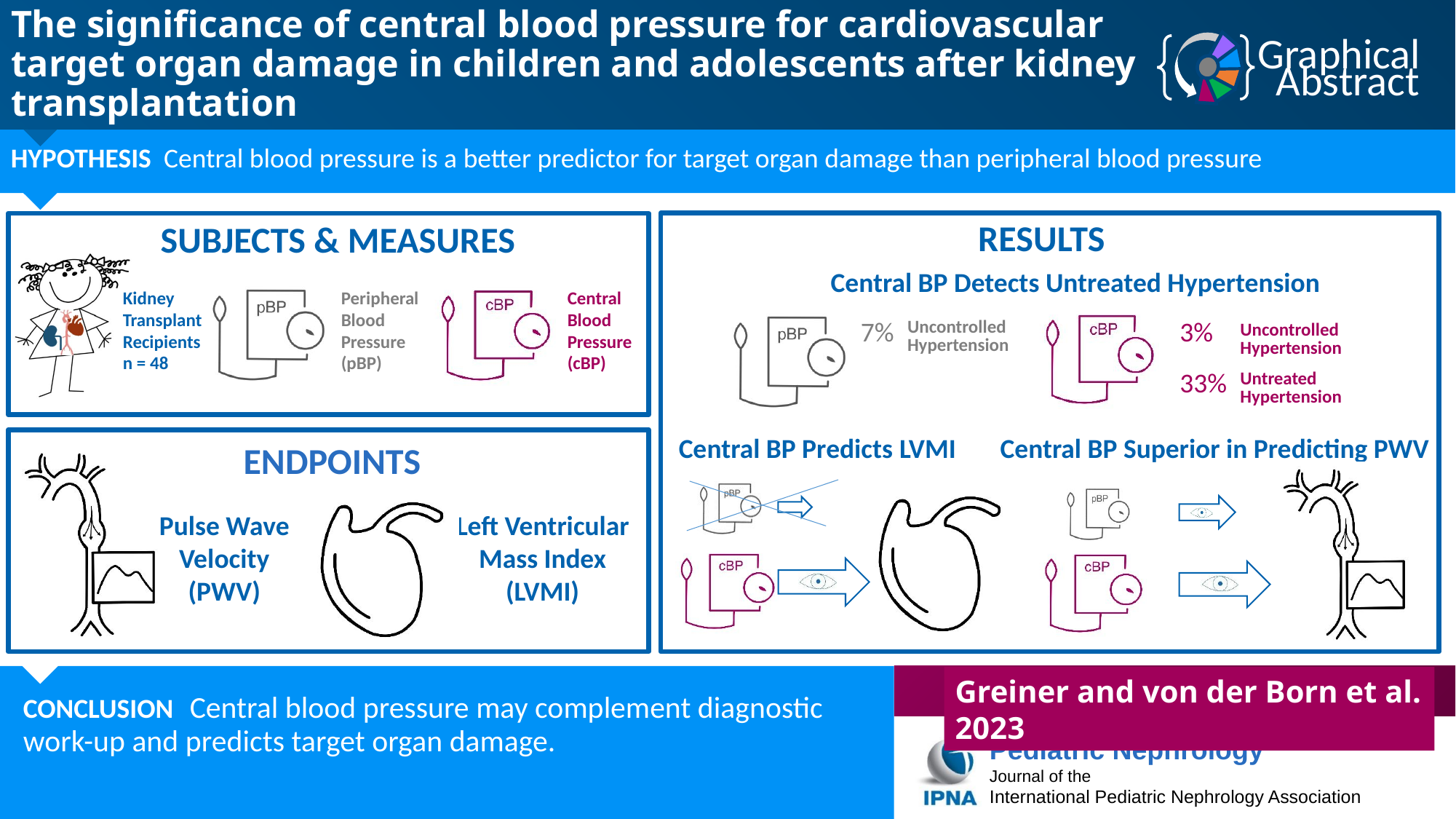

The significance of central blood pressure for cardiovascular target organ damage in children and adolescents after kidney transplantation
HYPOTHESIS Central blood pressure is a better predictor for target organ damage than peripheral blood pressure
Results
Subjects & Measures
 Central BP Detects Untreated Hypertension
Kidney Transplant Recipients
n = 48
Peripheral Blood Pressure (pBP)
Central Blood Pressure (cBP)
 Uncontrolled
Hypertension
7%
3%
Uncontrolled
Hypertension
33%
Untreated
Hypertension
Central BP Predicts LVMI
Central BP Superior in Predicting PWV
Endpoints
Pulse Wave Velocity
(PWV)
Left Ventricular Mass Index
(LVMI)
Greiner and von der Born et al. 2023
CONCLUSION Central blood pressure may complement diagnostic
work-up and predicts target organ damage.
